# Supplementary material for: Identification of photoperiod-induced specific miRNAs in the adrenal glands of Sunite sheep (Ovis aries)
Source: Front Vet Sci. 2022 Jul 22;9:888207. doi: 10.3389/fvets.2022.888207 (PMC9354845; doi:10.3389/fvets.2022.888207)
Supplement: Supplementary file 1 [file Data_Sheet_1.ZIP › Supplementary file/miRDeep2 file.zip!/miRDeep2 file/Know_Structure/LP42A3/oar-let-7b.pdf]

miRBase precursor : oar-let-7b
Total read count : 174405
oar-let-7b read count : 173756
remaining reads : 649

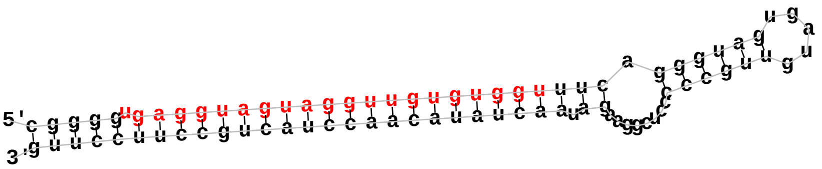

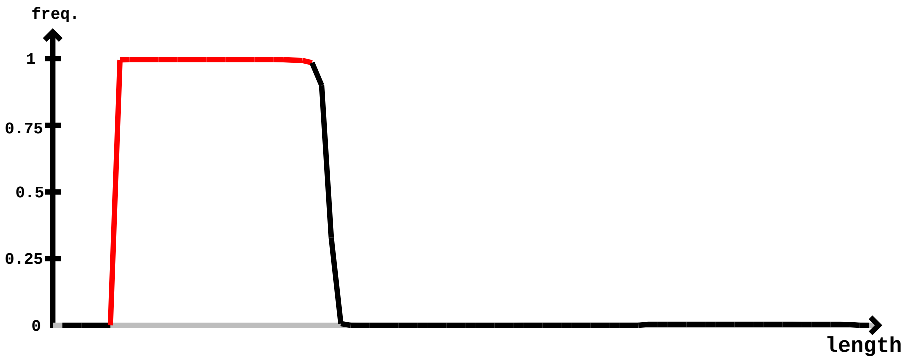


```
       oar-let-7b
5'- cggggugagguaguagguuguguggguuucaggguagugauguugcccccucggaagauaacuauacaaccuacugccuuccuug   -3'    exp
    (((((.(((((((((((((((((((((((.((((((.....))))))(....)..)).))))))))))))))))))))))))))    reads      mm         sample
    ....gugagguaguagguugugugguu.........................................................    2          0          seq
    .....ugagguaguagguugug..............................................................    25         0          seq
    .....ugaCguaguagguugug..............................................................    3          1          seq
    .....ugagguaguaAguugug..............................................................    1          1          seq
    .....ugagguaguagguugugu.............................................................    282        0          seq
    .....ugagguaguagguugugug............................................................    236        0          seq
    .....ugagguaguagguuguguggg..........................................................    1490       0          seq
    .....ugagguaguagguuguguggu..........................................................    14776      0          seq
    .....ugagguaguagguugugugguu.........................................................    99484      0          seq
    .....ugagguaguagguugugugguuu........................................................    56243      0          seq
    .....ugagguaguagguugugugguuuc.......................................................    1097       0          seq
    .....ugagguaguagguugugugguuuca......................................................    5          0          seq
    ......gagguaguagguuguguggu..........................................................    5          0          seq
    ......gagguaguagguugugugguu.........................................................    73         0          seq
    ......gagguaguagguugugugguuu........................................................    29         0          seq
    ......gagguaguagguugugugguuuc.......................................................    2          0          seq
    .......agguaguagguugugugguu.........................................................    2          0          seq
    ........gguaguagguugugugguu.........................................................    1          0          seq
    ...........................ucaggguagugauguugcccccucgga..............................    3          0          seq
    ...........................ucaggguagugauguugcccccucggaagau..........................    2          0          seq
    ...........................ucaggguagugauguugcccccucggaagaua.........................    2          0          seq
    .............................................ccccucggaagauaacuauacaac...............    1          0          seq
    ...............................................ccucggaagauaacuauaca.................    1          0          seq
    ....................................................gaagauaacuauacaaccuacug.........    1          0          seq
    .....................................................aagauaacuauacaaccuacugc........    2          0          seq
    ......................................................agauaacuauacaaccuacug.........    2          0          seq
    ......................................................agauaacuauacaaccuacugccuu.....    1          0          seq
    .........................................................uaacuauacaaccuacugccuuc....    1          0          seq
    .........................................................uaacuauacaaccuacugccuuccu..    1          0          seq
    ..........................................................aacuauacaaccuacugccuucc...    1          0          seq
    ............................................................cuauacaaccuacugccuu.....    2          0          seq
    ............................................................cuauacaaccuacugccuuc....    8          0          seq
    ............................................................cuauacaaccuacugccuucc...    105        0          seq
    ............................................................cuauacaaccuacugccuuccu..    486        0          seq
    ............................................................cuauacaaccuacugccuuccuu.    25         0          seq
```

oar-let-7b

```
cggggugagguaguagguugugugguuucaggguagugauguugcccccucggaagauaacuauacaaccuacugccuuccuug
.............................................................uauacaaccuacugccuucc...    1          0          seq
.............................................................uauacaaccuacugccuuccu..    4          0          seq
```
